# Core Endurance in University-Level Fast Bowlers: A Cross-Sectional Study

**DOI:** 10.7759/cureus.91204

**Published:** 2025-08-28

**Authors:** Ram Krishnan P, Geetha Sudha, Sai Aditya Raman, Arun C, Jibu George Varghese, Kedin Alwar Thiagarajan

**Affiliations:** 1 Sri Ramachandra Faculty of Sports and Exercise Sciences, Sri Ramachandra Institute of Higher Education and Research, Chennai, IND; 2 Arthroscopy and Sports Medicine, Sri Ramachandra Institute of Higher Education and Research, Chennai, IND; 3 Center for Sports Science, Sri Ramachandra Institute of Higher Education and Research, Chennai, IND

**Keywords:** asymmetry, core endurance, fast bowling, injury prevention, mcgill test, spinal stability

## Abstract

Background and objective

Fast bowling in cricket involves repetitive, high-velocity trunk movements that increase lumbar spine stress, making core endurance critical for spinal stability, bowling efficiency, and injury prevention. This study aimed to assess core endurance and lateral asymmetries in university-level fast bowlers.

Methods

Forty-two healthy male university fast bowlers (mean age: 20.19 ± 1.50 years), including 39 right-arm (92.9%) and three left-arm (7.1%) bowlers, who trained ≥3 times per week, participated in the study. The McGill Torso Endurance Test was employed to evaluate flexor, extensor, and lateral core endurance. Hold times were recorded, and descriptive statistics with paired and independent t-tests analyzed asymmetries.

Results

Mean core endurance times were 103.86 ± 23.91 seconds (flexor), 56.66 ± 11.94 seconds (extensor), 92.49 ± 26.79 seconds (right plank), and 79.56 ± 23.19 seconds (left plank). Compared to McGill norms (144 seconds, flexor; 146 seconds, extensor), participants showed reduced endurance, especially posteriorly. A significant lateral asymmetry was found, with 38 bowlers (90.5%) exhibiting longer hold times on the dominant side (mean difference = 12.92 seconds; p<0.001). Right-arm bowlers had greater right-side endurance, while the numerically smaller left-arm subgroup showed an opposite trend, though the differences were not statistically significant under unequal variance assumptions.

Conclusions

University-level fast bowlers demonstrated notable deficits in core endurance and significant side-to-side asymmetry favoring the dominant side, likely reflecting sport-specific adaptations. These findings underscore the need for targeted core conditioning, including unilateral training, to enhance spinal stability, reduce injury risk, and support bowling performance.

## Introduction

Cricket is a globally recognized bat-and-ball sport, deeply rooted in tradition and continually evolving through various competitive formats. Played between two teams of 11 players each on an oval field, the central focus is a 22-yard (20-meter) pitch with a wicket at each end, consisting of three stumps and two bails [1]. Among the key components of the game, bowling stands out as a primary skill and is broadly categorized into spin and fast bowling. Fast bowling is particularly defined by its combination of speed, accuracy, and strategic acumen. Unlike spin bowling, which relies on ball rotation and deception, fast bowling demands not only high delivery velocities but also tactical intelligence to exploit pitch conditions, environmental variables, and batsman weaknesses.

Biomechanically, fast bowlers are classified into front-on, side-on, and mixed-action types based on their posture and alignment during back foot contact. Each style imposes specific mechanical loads on the body and directly influences performance efficiency and susceptibility to injury [2]. Fast bowling is characterized by the generation of high ground reaction forces, rapid trunk rotation, and substantial kinetic energy transfer through the body, placing significant strain on the musculoskeletal system - particularly the lumbar spine. The performance of fast bowlers is thus dependent on physical qualities such as strength, coordination, flexibility, and neuromuscular control [3,4]. The complexity of the bowling action underscores the need for biomechanical efficiency and physical conditioning to prevent injury and enhance performance.

A key component of this conditioning is core endurance, which refers to the capacity of the core musculature - including the abdominals, back extensors, hips, and pelvis - to maintain postural control and resist fatigue during prolonged or repetitive physical activity [5,6]. For fast bowlers, core endurance is vital for stabilizing the spine under repetitive loads, preserving technique over prolonged spells, and reducing injury risk [7]. Core endurance plays a central role in maintaining spinal alignment during the delivery stride and supports efficient energy transfer through the kinetic chain. The McGill Torso Endurance Test is a standardized and validated assessment protocol designed to evaluate muscular endurance of the torso through three isometric tests targeting the anterior, posterior, and lateral musculature. These include the flexor endurance test (targeting rectus abdominis and obliques), the extensor endurance test (targeting erector spinae and multifidus), and the lateral endurance or side bridge test (targeting quadratus lumborum, obliques, and transverse abdominis)[8]. Widely used in clinical and sports performance settings, these tests offer objective metrics to evaluate core endurance and spinal stability, which are critical in high-load sports such as fast bowling.

Due to the high-intensity and repetitive nature of the bowling action, fast bowlers are at increased risk of lumbar spine degeneration, with lumbar spondylosis being a commonly reported condition [9,10]. This degenerative pathology involves the deterioration of intervertebral discs, facet joints, and vertebral structures, often leading to disc space narrowing, facet joint degeneration, and segmental instability [11]. Epidemiological studies report that abnormalities of the lumbar spine, including spondylolysis, affect between 24% and 55% of adult fast bowlers. These injuries result from chronic axial compression, torsional stress, and repetitive flexion-extension cycles, often manifesting as lower back pain, stiffness, and functional limitations [12].

Given these biomechanical demands and associated injury risks, the assessment of core endurance in fast bowlers is crucial. The present study aims to assess the torso endurance of university-level fast bowlers using the McGill Torso Endurance Test, with a specific focus on identifying asymmetries between the dominant and non-dominant sides. By addressing a critical gap in the literature, this study seeks to provide data on current torso endurance levels and asymmetrical core strength patterns among fast bowlers [10,13]. We believe the findings will help inform injury prevention strategies and guide the development of evidence-based rehabilitation, strength and conditioning, and performance enhancement programs tailored for fast bowlers.

## Materials and methods

This cross-sectional observational study was conducted at the Sri Ramachandra Centre for Sports Science, Sri Ramachandra Institute of Higher Education and Research (Deemed to be University), Chennai, over a period of three months from December 2024 to February 2025. The study included 42 male university-level fast bowlers (100%). The study aimed to assess core endurance in university-level fast bowlers. The sample size was determined based on a previous study 14, which reported a mean flexor endurance score of 110.5 ± 54.55 seconds among fast bowlers. With a relative precision of 15% and a 95% confidence level, the required sample size was calculated as 42 and was rounded up to 45 to accommodate potential dropouts. Participants were male university-level fast bowlers actively engaged in competitive or institutional cricket programs.

Inclusion criteria required participants to be within the age range of 18-26 years, with no history of lower back, pelvic, or hip injuries in the past six months, and no major surgeries in the last two years that could affect physical performance. Additionally, eligible participants had to engage in cricket training at least three times per week to ensure consistent exposure to fast bowling mechanics. Participants were excluded if they had any current or recent injuries involving the lower back, pelvis, or hips; had undergone major surgical procedures in the past two years; were outside the defined age range; or trained less than three times per week. All participants provided informed consent before participation in the study.

Statistical analyses

Multiple statistical tests were employed in this study to analyze the data comprehensively. Descriptive statistics (mean ± SD) were used to summarize demographic characteristics and endurance test results. To assess data distribution, normality tests (Kolmogorov-Smirnov and Shapiro-Wilk) were performed, confirming the use of parametric tests. A paired-samples t-test was conducted to compare left and right side plank times, identifying intra-individual asymmetry. An independent-samples t-test (with Levene's test for equality of variances and the Welch correction as appropriate) was used to compare asymmetry patterns between left- and right-arm bowlers. Additionally, Pearson correlation analysis was used to explore the relationship between left and right plank durations. All tests were conducted using a two-tailed significance level of 0.05.

Recruitment and materials

Participants were recruited either through in-person meetings or online sessions, during which the investigator explained the purpose and procedures of the study in detail to both the individual players and their respective team coaches. Those who voluntarily agreed to participate were asked to provide written informed consent before enrollment. After obtaining consent, each participant completed a structured questionnaire capturing demographic information, training history, and any prior injury history. Based on the analysis of these responses, only those individuals who met the inclusion criteria were selected for the study. The materials used for the assessment included a stopwatch with a precision of 0.01 seconds for timing, a test bench, and wedges to assist in standardized positioning during the extensor and flexor components of the test, and yoga mats for participant comfort and stability during the lateral (side plank) test. The McGill Torso Endurance Test was subsequently administered to all eligible participants.

Methodology

The McGill Torso Endurance Test, comprising three components - the flexor endurance test, extensor endurance test, and side plank test - was employed to assess core muscular endurance in university-level fast bowlers. Out of 42 fast bowlers, 39 (92.9%) were right-arm and three (7.1%) were left-arm bowlers.. The flexor endurance test evaluates anterior core endurance. Participants were positioned with their hips and knees flexed at 90 degrees, reclining against a 60-degree inclined wedge with their feet secured. Arms were crossed over the chest, and once the wedge was removed, participants were instructed to maintain the inclined posture unsupported for as long as possible without compromising form [15]. The extensor endurance test measures posterior core endurance. Participants lay prone on a test bench with their iliac crests aligned at the edge of the bench. A helper supported their upper body in a horizontal position until the start of the test. Upon initiation, the support was removed, and participants were required to hold the horizontal posture unassisted for as long as possible [15]. The side plank test, performed on both right and left sides, assesses the endurance of lateral core muscles [16]. Participants were positioned on their side, propped on one elbow and the side of the foot, with the non-supporting arm crossed over the chest. They were instructed to lift their hips off the ground and maintain a straight, aligned posture for as long as possible. Each side was tested separately, and the duration for which the position was held was recorded [15]. All tests were conducted under standardized conditions, with verbal encouragement provided to ensure maximal effort.

## Results

Table 1 presents the descriptive statistics of the study participants (N = 42), comprising university-level fast bowlers with a mean age of 20.19 years (SD = 1.50), an average height of 173.12 cm (SD = 6.41), and a mean body weight of 68.38 kg (SD = 8.25).

**Table 1:** Descriptive statistics SD: standard deviation

Variable	Mean	SD
Age	20.19	1.502
Height (cm)	173.12	6.406
Weight (kg)	68.38	8.249

Table 2 details the distribution of bowling arm dominance, revealing that the majority of participants were right-arm bowlers (n = 39, 92.9%), while a small minority were left-arm bowlers (n = 3, 7.1%).

**Table 2:** Distribution of left- and right-handed bowlers

Bowling arm	Frequency	Percent
Left	3	7.1
Right	39	92.9
Total	42	100.0

**Figure 1:**
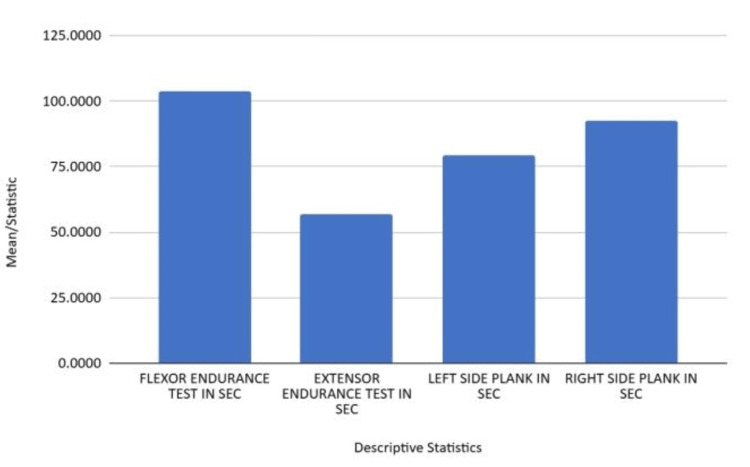
Core endurance performance across flexor, extensor, and lateral plank tests

 Figure 1 demonstrates the core endurance performance across flexor, extensor, and lateral plank tests.

Table 3 summarizes the mean values and standard deviations for each component of the McGill Torso Endurance Test. The flexor endurance test had an average duration of 103.86 seconds (SD = 23.91), the extensor endurance test averaged 56.66 seconds (SD = 11.94), the left side plank averaged 79.56 seconds (SD = 23.19), and the right side plank averaged 92.49 seconds (SD = 26.79), indicating variability in core endurance across muscle groups and evidence of lateral asymmetry.

**Table 3:** Core endurance test results among university-level fast bowlers SD: standard deviation

Test	Mean	SD
Flexor endurance test (s)	103.8571	23.90690
Extensor endurance test (s)	56.6619	11.94195
Left side plank (s)	79.5610	23.18916
Right side plank (s)	92.4850	26.78945

Table 4 outlines the results of the Shapiro-Wilk normality test, with all components of the McGill test showing non-significant p-values (p>0.05), thereby confirming the assumption of normality and justifying the use of parametric inferential tests.

**Table 4:** Tests of normality

Test	Kolmogorov-Smirnova			Shapiro-Wilk		
	Statistic	Df	Sig.	Statistic	Df	Sig.
Flexor endurance test	0.123	42	0.114	0.959	42	0.140
Extensor endurance test	0.107	42	0.200	0.985	42	0.859
Left side plank	0.113	42	0.200	0.971	42	0.364
Right side plank	0.118	42	0.158	0.970	42	0.331

**Figure 2:**
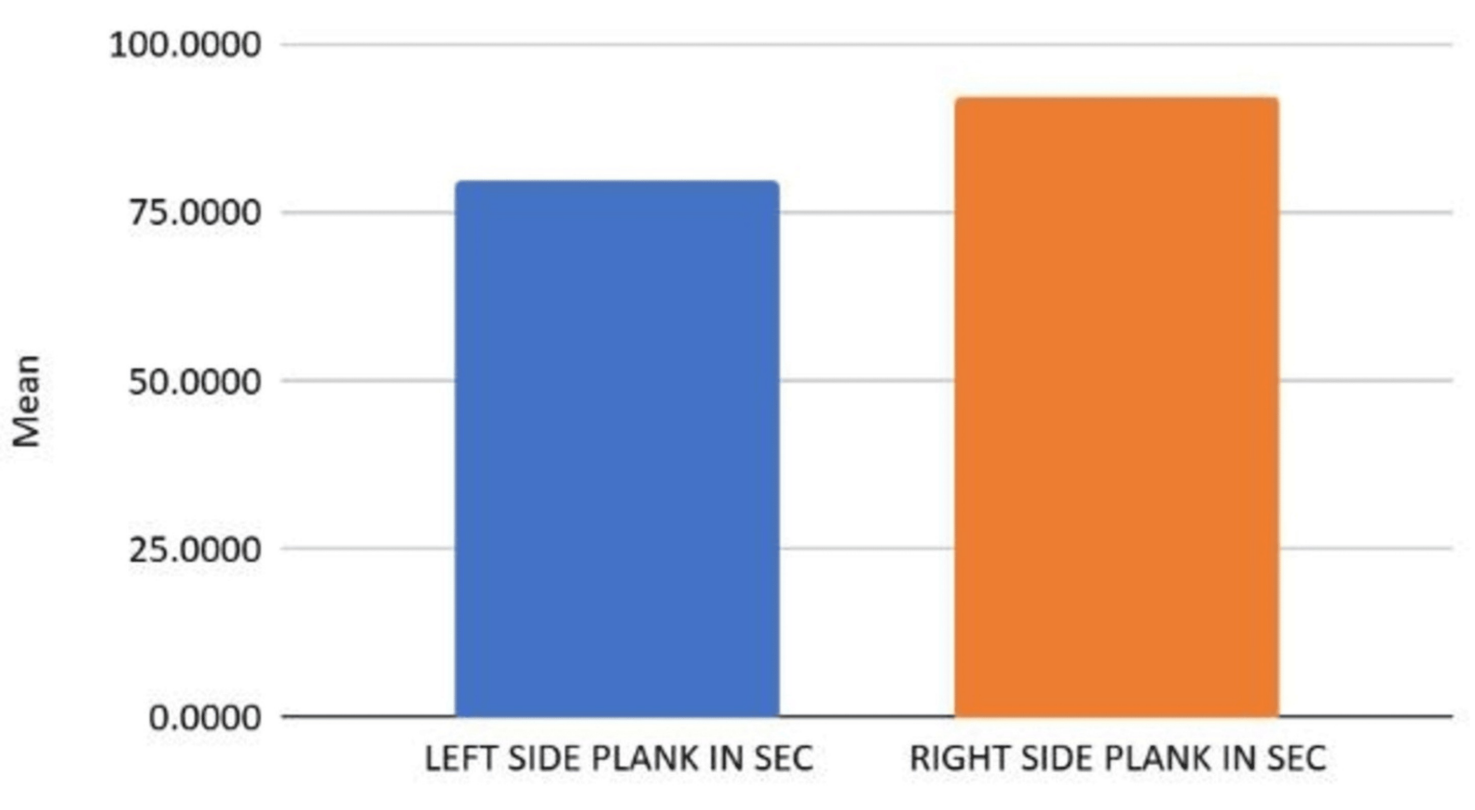
Asymmetry in lateral core endurance between the left and right sides

Figure 2 illustrates the asymmetry in lateral core endurance between the left and right sides.

Table 5 provides the results of the paired samples t-test comparing left and right side plank performance, which revealed a statistically significant difference (t(41) = -6.297, p<0.001), with greater endurance observed on the right side (M = 92.49 sec) compared to the left (M = 79.56 sec). The mean difference of -12.92 seconds highlights a consistent lateral asymmetry favoring the right side. A strong positive correlation (r = 0.868, p<0.001) was also noted between left and right plank durations, suggesting a shared performance trend despite the dominance-related disparity.

**Table 5:** Comparison of side plank asymmetry between left- and right-arm bowlers

			Levene's test for equality of variances	T-test for equality of means	
		F	Sig.	T	Sig. (2-tailed)
Plank diff	Equal variances assumed	4.788	0.035	-4.367	0.000
	Equal variances not assumed			-2.253	0.149

**Figure 3:**
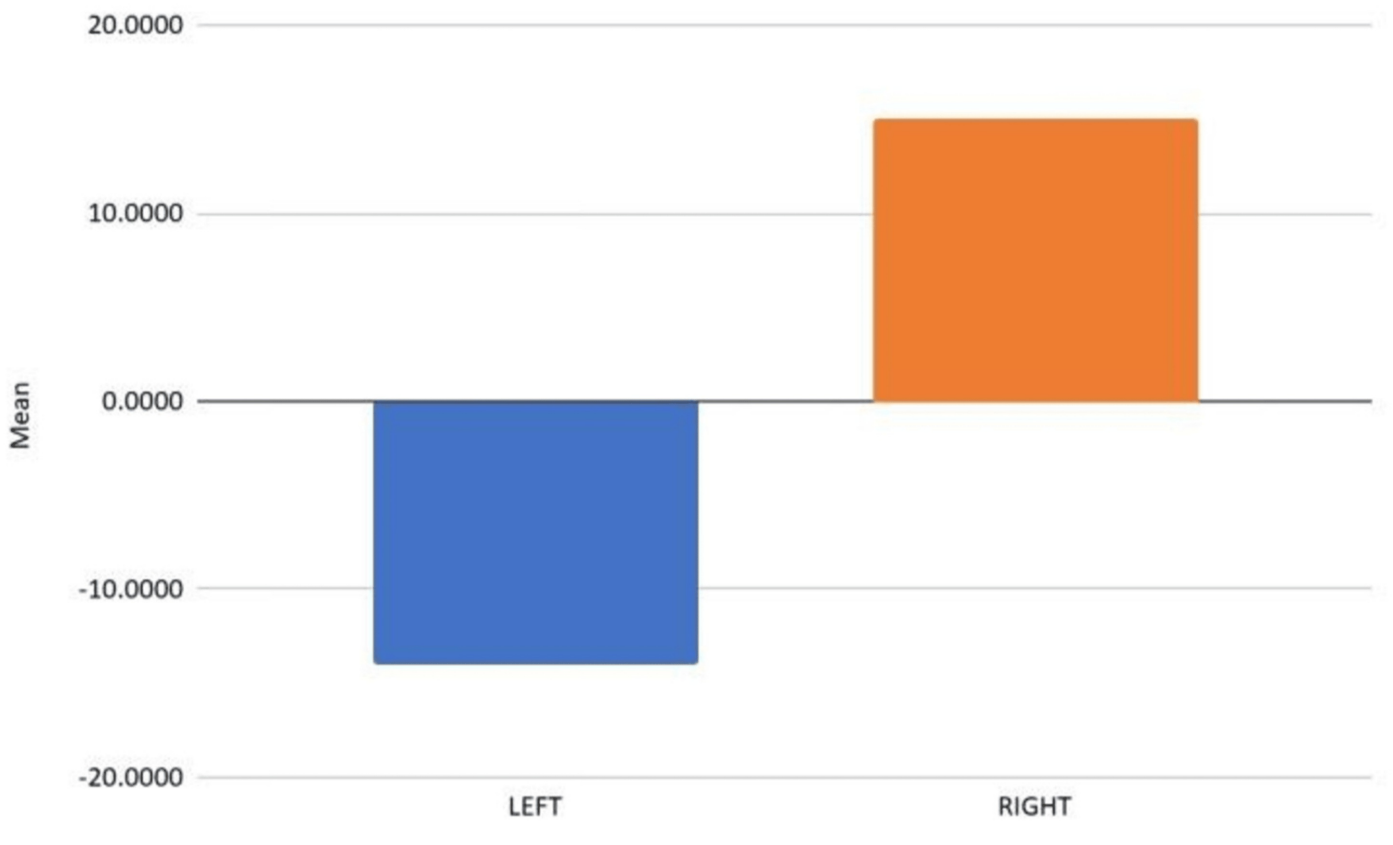
Variation in side plank asymmetry between left- and right-arm fast bowlers Mean vs. paired samples statistics

 Figure 3 depicts the variation in side plank asymmetry between left- and right-arm fast bowlers.

## Discussion

In the context of high-performance cricket, fast bowling represents a biomechanically demanding movement pattern that places substantial emphasis on core stability and muscular endurance to support ball velocity, precision, and injury prevention. The current study was designed to evaluate core muscular endurance among university-level fast bowlers, recognizing that repeated high-velocity trunk motions inherent in bowling demand sustained muscular control for optimal kinetic chain efficiency and spinal protection [17,18]. Insufficient core endurance or the presence of muscular imbalances may compromise bowling mechanics, reduce energy transfer efficiency, and increase susceptibility to overuse injuries, particularly within the lumbar spine [19,20]. This investigation specifically aimed to quantify anterior, posterior, and lateral core endurance and to analyze any asymmetry between dominant and non-dominant sides that may contribute to biomechanical inefficiency or injury risk.

The findings provide foundational insight into the core endurance profiles of young male fast bowlers. Participants were predominantly right-arm dominant (n = 39, 92.9%), reflecting common hand dominance trends in athletic populations. This distribution bears clinical relevance, as sports involving repetitive unilateral trunk rotation - such as fast bowling - may predispose athletes to side-specific adaptations and compensatory movement strategies [21-23]. During bowling delivery, right-arm bowlers typically generate a left-to-right rotational torque, which may place greater strain on the non-dominant lateral stabilizers to maintain trunk integrity.

The endurance data derived from the McGill Torso Endurance Test demonstrated varying levels of muscular capacity across core regions. Flexor endurance, reflective of anterior stability, showed a mean holding time of 103.86 ± 23.91 seconds, with substantial variability ranging from 52 to 143 seconds, suggesting that while the group generally possessed moderate endurance capacity, individual differences - likely influenced by training history, biomechanics, and prior injury - were pronounced. Posterior endurance, assessed via the extensor test, was markedly lower, with a mean duration of 56.66 ± 11.94 seconds and the lowest recorded value at 26.42 seconds. These findings suggest potential underdevelopment of the spinal extensors or early fatigue onset in this muscle group, a concern given the repetitive lumbar extension and axial loading involved in fast bowling [24].

The most prominent finding was the significant lateral asymmetry in core endurance, with right-side planks showing a higher mean duration (92.49 ± 26.79 seconds) compared to the left (79.56 ± 23.19 seconds). Given that the majority of participants were right-arm bowlers, this discrepancy likely reflects chronic unilateral loading patterns during the delivery phase. The repetitive recruitment of dominant-side stabilizers may result in side-specific hypertrophy or endurance adaptations [21,22]. This imbalance, if unaddressed, can lead to altered spinal mechanics and increase the risk of overuse injuries over time [25].

To contextualize these endurance profiles, the results were compared with normative values reported by McGill et al., where healthy adult males demonstrated mean values of 144 seconds (flexor), 146 seconds (extensor), and approximately 94 and 97 seconds for the right and left side bridges, respectively [26]. The current study's participants fell well below these norms, particularly for posterior chain endurance, which was less than 40% of the benchmark. This discrepancy suggests a notable deficit in posterior and lateral core endurance, potentially due to training limitations, biomechanical stress, or lack of emphasis on specific trunk stabilization exercises within cricket conditioning routines.

The side plank results also revealed a lateral endurance ratio well below the normative symmetry threshold. While the right side approached normative levels, the left was significantly weaker, highlighting a pronounced dominance effect. Paired sample t-test analysis confirmed a statistically significant difference favouring the dominant (right) side. Notably, a strong positive correlation (r = 0.868, p<0.001) was observed between left and right side plank durations, indicating that individuals with better endurance on one side generally performed better overall. Nonetheless, this persistent imbalance may have functional consequences, particularly if not addressed in injury prevention or performance programs.

To explore the influence of arm dominance on endurance asymmetry, further analysis compared lateral endurance differences between left- and right-arm bowlers. Results indicated a trend toward higher endurance on the dominant side in both groups. However, due to the very small number of left-arm bowlers (n = 3), the statistical power of this comparison was limited. While the initial analysis assuming equal variances suggested a significant difference (p<0.001), Levene’s test indicated unequal variance, and the more robust analysis under “equal variances not assumed” revealed a non-significant result (p = 0.149). As such, the observed asymmetry may be meaningful, but these findings must be interpreted with caution due to sample size constraints.

These observations align with findings from previous research on trunk muscle asymmetry [22]. For instance, identified asymmetries in abdominal muscle thickness in fast bowlers, noting greater thickness in the non-dominant external oblique, and a moderate association between external oblique symmetry and ball release speed. While the present study focused on functional asymmetry (muscle endurance), both investigations underscore the relevance of lateral trunk balance in performance and injury prevention. Importantly, these results highlight that not all asymmetries are inherently maladaptive - some may represent sport-specific functional adaptations [27].

In summary, this study identified significant differences in anterior, posterior, and especially lateral core endurance among university-level fast bowlers, with clear dominance-related asymmetries. These findings underscore the importance of targeted core training that addresses both overall endurance and side-to-side balance. Future research with larger, more evenly distributed samples and longitudinal tracking may further clarify the impact of these asymmetries on performance and injury risk. Until then, cricket training programs should consider integrating corrective and unilateral core strengthening strategies to promote muscular balance and mitigate injury potential.

Limitations

This study has several limitations that should be acknowledged. First, the unequal sample distribution, specifically the small number of left-arm bowlers (n = 3, 7.1%), reduced the statistical power to detect differences based on arm dominance and limits the generalizability of findings in this subgroup. Second, the cross-sectional design restricts the ability to infer causality or to assess how core endurance evolves with training, adaptation, or over the course of a competitive season. Third, the study exclusively employed static endurance tests without incorporating biomechanical analyses or electromyographic (EMG) assessments, which would have offered more nuanced insights into muscular recruitment patterns and dynamic trunk function during cricket-specific movements. Additionally, the participant pool included only male university-level bowlers, limiting the applicability of results to female athletes and younger or elite populations. Lastly, the absence of direct performance metrics such as ball velocity, bowling accuracy, or injury occurrence precludes a comprehensive evaluation of the practical implications of core endurance and asymmetry on athletic performance or injury risk.

Future research directions

Future studies should seek to recruit a more balanced sample in terms of bowling arm dominance and include female and adolescent bowlers to enhance external validity. Longitudinal designs are recommended to explore how core endurance and asymmetry respond to targeted interventions or training regimens over time. The integration of biomechanical tools such as EMG, 3D motion analysis, or wearable technology could provide functional insights into muscle activation patterns during dynamic phases of the bowling action. Furthermore, correlating core endurance measures with objective performance outcomes (e.g., ball speed, bowling efficiency) and injury surveillance data would help bridge the gap between laboratory findings and field applications. Such evidence could guide the development of sport-specific conditioning programs tailored to the demands of fast bowling.

## Conclusions

The present study highlights the important role of core muscular endurance, particularly lateral endurance, in the performance and injury prevention of university-level fast bowlers. The findings show notable deficits in extensor and flexor endurance when compared to established normative values, as well as a significant asymmetry in lateral endurance, favoring the dominant side. The occurrence of this imbalance likely reflects sport-specific adaptations resulting from the repetitive, unilateral demands of fast bowling. While some degree of asymmetry may be functionally advantageous, the extent observed in this cohort suggests a potential risk for biomechanical inefficiency and injury over time. These results underscore the need for targeted conditioning programs that not only enhance global core endurance but also correct side-to-side imbalances through unilateral training and functional rehabilitation. Future research with larger, more diverse cohorts and longitudinal designs is warranted to further explore the long-term impact of core endurance asymmetry on performance outcomes and injury incidence in fast bowlers.
